# 

**Published:** 1896-11

**Authors:** 


### PERSONAL.

Dr. H. J. Brotheridge, of Brooklyn, was a recent visitor to the JOURNAL office.

Dr. H. B. Adair received an appointment in the meat-inspection service in the Bureau of Animal Industry, and has been stationed at Milwaukee.

Dr. J. C. Milnes, of Cedar Rapids, Iowa, and Dr. Don Patten, of Hampton, Iowa, have received appointments as meat-inspectors, and have been assigned to duty at Kansas City.

Dr. A. D. Melvin has recently made a tour of the large cities where government meat-inspection is being conducted, his chief purpose aiming to make the inspection at all points as uniform as possible.

Dr. James B. Paige, of Amherst, Mass., has returned to his field of labor after a profitable sojourn abroad.

Veterinarian Edward Crandall was consulting veterinary surgeon to the annual horse-show of the Genesee Valley Club, at Geneseo, N. Y.

Dr. J. McBirney, recently stationed at Philadelphia as an inspector in the Bureau of Animal Industry, has been transferred to Chicago.

Veterinarian G. Howard Davison, of Millbrook, N. Y., has been elected Secretary of the National Association of Exhibitors of Live-stock at the third annual meeting at Springfield, Ill., in September.

Veterinarians and their subjects before the Farmers' Institutes of Pennsylvania during the coming institute year:

Dr. M. E. Conrad, V.M.D., West Grove, Chester Co., Pa.: The Horse's Foot and Its Treatment; How to Replenish Our Dairy Herds; Breeding and Care of Farm Horses; Diseases of Dairy Cattle; Tuberculosis.

Dr. J. B. Irons, V.S., Erie, Erie Co., Pa.: Tuberculosis in Cows Causing Consumption in Men; Hollow Horn; Lost Cud; Wolf in Tail, and Treatment; Acute Indigestion in the Horse and How to Distinguish it from Other Forms of Colic, and Treatment.

Dr. C. C. McLean, Meadville, Pa.: Diseases Causing Lameness; Dairy Sanitation; Diseases of Milch Cows; Talk on any Horse Disease Requested.

Dr. Leonard Pearson, Harrisburg, Pa.: How to Keep Farm Animals Healthy; Other Topics on Application.

Dr. James Waugh: Diseases of the Digestive Organs of the Horse; Tuberculosis Among Our Domestic Animals; Facts About Milk-fever in Fresh Cows; Modern Live-stock Breeding for Profit; Influence of Character and Force of Habit in Mankind.

Dr. W. Horace Hoskins: Some of the Avenues Through which the Veterinarian may Contribute to the Success of Agriculture; Some of the Dangers Incidental to Milk-production as a Raw-food Product; The Value of Good Roads from a Veterinary Point of View.

Dr. W. H. Dalrymple, of Baton Bouge, La., addressed the North Louisiana Agriculture Association on September 18th, on the proper care of stock. In his address he referred to the standing of the veterinary profession in the North, and of the welcome of the U. S. V. M. A. at Buffalo by the city officials, the veterinary profession, and of the people in general, and expressed the hope that the South might extend such a generous invitation to the Association should it hold its meeting of 1897 in that territory.

Prof. Andrew Smith was recently elected a director of the Toronto (Canada) County Hunt Club.

Dr. J. Stewart Lacock has just returned from a trip to Chicago, Milwaukee, and Cleveland.

Dr. J. C. McNeil, of Pittsburg, Pa., was a recent visitor to the JOURNAL office.

Dr. R. S. Huidekoper will be the attending veterinarian of the live-stock show at the Madison Square Garden on November 23d to 28th.

Veterinarian A. S. Alexander, of Evanston, Ill., will be the judge of Aberdeen-Angus cattle at the live-stock show in New York in November.

Governor Morton has appointed as the examiners, under the recent act passed by the Legislature governing the future horseshoers of New York State, Veterinarian Thomas M. Quinn; of the mastershoers, Thomas Carroll, of New York, and Robert Keenan, of Brooklyn; and as journeymen horseshoers, Homer A. Grove, of Rochester, and Charles W. Kirk, of Albany.

Dr. A. W. Althouse recently brought suit for the sum of $865 for professional services against C. J. Hamlin, of Buffalo, in the circuit court of Lexington, Ky.

Dr. M. P. Ravenel has been appointed director and bacteriologist at the new veterinary laboratory founded by the State Live-stock Sanitary Board at the Veterinary Department of the University of Pennsylvania.

Dr. S. J. J. Harger, of Philadelphia, has been on the sick-list, though he courageously attended to his duties as Secretary of the Pennsylvania State Board of Veterinary Medical Examiners on October 20th and 21st at Harrisburg, Pa.

